# Insights on E1-like enzyme ATG7: functional regulation and relationships with aging-related diseases

**DOI:** 10.1038/s42003-024-06080-1

**Published:** 2024-03-29

**Authors:** Jingwei Liu, Yutong Xiao, Liangzi Cao, Songming Lu, Siyi Zhang, Ruohan Yang, Yubang Wang, Naijin Zhang, Yang Yu, Xiwen Wang, Wendong Guo, Zhuo Wang, Hongde Xu, Chengzhong Xing, Xiaoyu Song, Liu Cao

**Affiliations:** 1 The College of Basic Medical Science, Health Sciences Institute, China Medical University, Shenyang, Liaoning China; 2 Key Laboratory of Cell Biology of Ministry of Public Health, Key Laboratory of Medical Cell Biology of Ministry of Education, Key Laboratory of Precision Diagnosis and Treatment of Gastrointestinal Tumors (China Medical University), Ministry of Education, Liaoning Province Collaborative Innovation Center of Aging Related Disease Diagnosis and Treatment and Prevention, China Medical University, Shenyang, Liaoning China; 3 Department of Anus and Intestine Surgery, First Affiliated Hospital of China Medical University, Shenyang, Liaoning China; 4 Department of Cardiology, First Hospital of China Medical University, Key Laboratory of Environmental Stress and Chronic Disease Control and Prevention, Ministry of Education, China Medical University, Shenyang, Liaoning China; 5 Department of Thoracic Surgery, Shengjing Hospital of China Medical University, Shenyang, Liaoning China

**Keywords:** Macroautophagy, Post-translational modifications

## Abstract

Autophagy is a dynamic self-renovation biological process that maintains cell homeostasis and is responsible for the quality control of proteins, organelles, and energy metabolism. The E1-like ubiquitin-activating enzyme autophagy-related gene 7 (ATG7) is a critical factor that initiates classic autophagy reactions by promoting the formation and extension of autophagosome membranes. Recent studies have identified the key functions of ATG7 in regulating the cell cycle, apoptosis, and metabolism associated with the occurrence and development of multiple diseases. This review summarizes how ATG7 is precisely programmed by genetic, transcriptional, and epigenetic modifications in cells and the relationship between ATG7 and aging-related diseases.

## Abstract (web summary)

A review summarizes how ATG7 is precisely programmed by genetic, transcriptional, and epigenetic modifications in cells and its functions, including the relationship between ATG7 and aging-related diseases.

## Introduction

Autophagy is essential for the degradation and reutilization of proteins^1^ and transforms various cytoplasmic cargo (e.g., microbes, organelles, and cytotoxic protein aggregates) into smaller molecules to energize cell metabolism and maintain the renewal of organelles^2^. During this process, the cytoplasmic components are isolated into double-membrane structures named autophagosomes, transported to lysosomes, and degraded^3^.

Aging is a popular topic in human health research. Although it is an unavoidable physiological process, it is also the spatiotemporal background of many diseases, including neurodegenerative diseases, diabetes, skeletal muscle atrophy, and tumors. Recent studies have shown that autophagy plays an important role in aging and the occurrence and development of aging-related diseases.

**Fig. 1:**
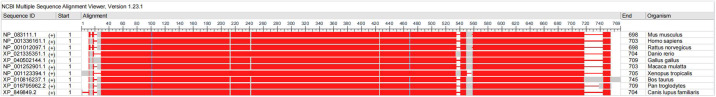
*Atg7* gene conservation among common species. *Atg7* is highly conserved among different species.

**Fig. 2:**
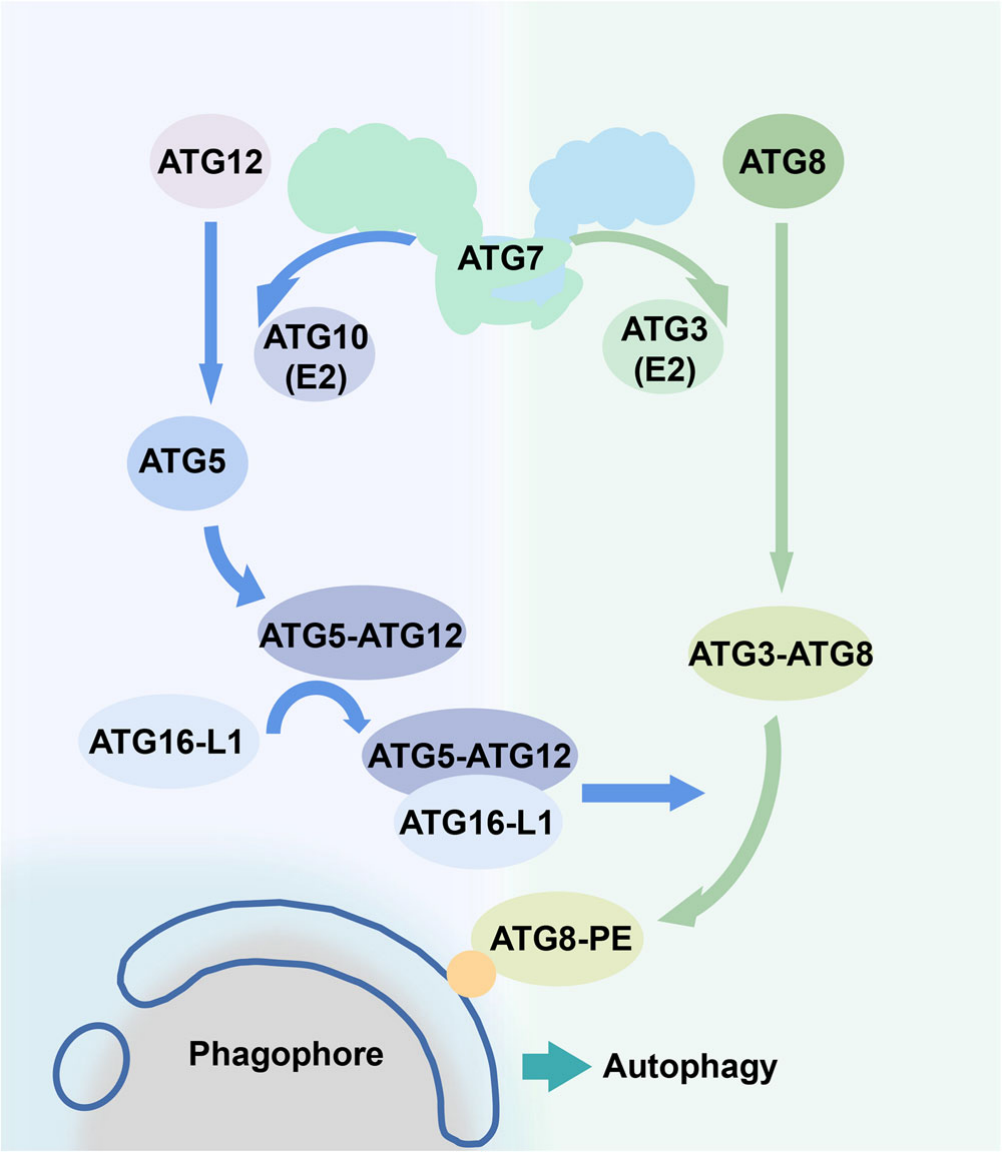
Classical autophagy process mediated by ATG7 as E1-like enzyme. ATG7 protein is a homodimeric E1 enzyme that mediates covalent modifications of other autophagy-related proteins during autophagy, including the binding of ATG5 and ATG12, and the lipidation of LC3.

Approximately 40 autophagy-related genes (*Atgs*) are part of the autophagy machinery. *Atg7* is an essential autophagy-related gene that encodes a series of proteins of ~630–700 residues in length found in various eukaryotes^4^. ATG7 is a homodimeric E1 enzyme that drives ATG8 and ATG12 to their own E2 enzymes, ATG3 and ATG10, respectively^5^. The 3D structure of the C-terminal domains of the dimer is shaped like the body of a bird, with the ATG-binding N-terminal domains similar to wings^5,6^. The interactions between ATG7 and the E2 core domain of ATG3 and ATG10 are markedly similar across different species^7^. ATG7 is also associated with multiple biological functions beyond the activation of autophagy^8^. We found that ATG7 modulates p53 activity to regulate the cell cycle and survival during metabolic stress. ATG7 also plays important roles in protein lipidation events similar to ubiquitination and membrane fusion events during autophagy^9^. This protein is critical to several processes, including the precaution of axonal neurodegeneration^10^, the preservation of hematopoietic stem cells^11^, and adipose differentiation^12^ (Figs. 1 and 2).

## Regulation on ATG7 and its relationship with aging-related diseases

### Regulation of the *Atg7* gene

#### *Atg7* variants and gene fusion

Previous studies in multiple *Atg7* knockout (KO) animal models investigated the relationship between *Atg7* and altered biological processes (Table 1). Several additional studies focused on genetic alterations in *Atg7* and their impact on aging-related diseases. A putative G-quadruplex-forming sequence folds into a four-stranded G-quadruplex (G4) structure in the first intron of *Atg7*. This structure always appears in guanine-rich DNA sequences, regulating gene replication and transcription in cancer cells and neurons^13^. G4-ligand pyridostatin can form 1:1 and 2:1 complexes with G4 in *Atg7*, inhibiting autophagy by stabilizing the G4-DNA complex^14^. Another distinct G4-DNA-binding ligand, BRACO19, can downregulate *Atg7* transcription. The formation and disassociation of *Atg7* G4-DNA is the original pathway for regulating autophagy in neurons, which correlates with the effect of age on memory^15^.

**Table 1:** Representative *Atg7*-KO animal models

Target	Description		Reference
Whole body	Lower mean body weight and earlier death, partly caused by the interrupt of amino acids recycling		^220^
	Disrupted cell cycle		^203^
	Enhanced HFD-mediated NRF2 downregulation, possibly influencing the degree of liver damage and the development of hepatic steatosis		^221^
	Enhanced macrophage activation and Concanavalin A-induced acute hepatitis		^222^
Viscus	Kidney	In inducible, renal tubule-specific *Atg7*-KO mice, the pro-fibrotic phenotype of tubular cells is induced. Meanwhile, fibrosis is down-regulated after AKI.	^223^
		Renal proximal tubule-specific *Atg7*-KO mice are significantly more sensitive to cisplatin-induced AKI.	^224^
	Liver	Loss of autophagic function inhibits lipid release and hepatic fibrogenesis in ATG7-interferred hepatic stellate cells.	^225^
	Intestine	The intestinal epithelial-specific ATG7 deficiency in mouse model has a negative influence on the regenerative benefit of calorie restriction, partly caused by the function of ATG7 in modulating luminal glycocholic acid, which is crucial to the self-renewal of epithelial stem cell.	^226^
		Unlike the oncogene role of ATG7 in colon cancer, the researches about inflammation-associated colon tumorigenesis in intestinal epithelial-specific *Atg7*-KO mice show a negative effect on tumorigenesis of colitis-associated cancer.	^227^
		Intestinal ATG7 deficiency causes intestinal dysbiosis, which leads to a suppression of tumor initiation and growth.	^228^
	Pancreas	Impaired glucose tolerance is observed in pancreatic β cell-specific *Atg7*-KO mice, along with the accumulation of p62, which is required for cellular homeostasis regulation.	^229^
		Researches in ATG7-deleted β-cells with both long-term and short-term HFD mouse model employed, exhibit that short-term ATG7 deletion benefits β-cell function and enhance glucose-stimulated insulin secretion, while the long-term is opposite.	^230^
	Lung	Silencing ATG7 in lung-to-lung metastasis mice can turn the reduction of tumor nodules and cancer cell metastasis caused by increasing TIMP1.	^231^
Tissue	Adipose	In adipocyte-specific *Atg7*-KO mice, HFD-induced inguinal white adipose tissue hypertrophy is promoted.	^219^
	Skin	In ATG7-negative keratinocytes, autophagy induced by UVA or UVA-oxidized phospholipids is interrupted, which further causes obstructed removal of protein aggregates and oxidized phospholipids.	^232^
		Researches carried out in ATG7-inactivated epidermal keratinocytes exhibits that corneocytes on the back of mutant mice is intensified.	^233^
		ATG7 deletion in EC assists skin wound healing via promoting paracrine regulation.	^234^
	Neuron	Excitatory forebrain neurons-specific ATG7 deletion in mouse model markedly inhibits Aβ secretion, which is the pathological character of AD.	^235^
		Mitochondrial hyperfusion is found in ATG7-deficient astrocytes, which plays a key role in cell fate specification during autophagy	^236^
	Muscle	In L6 skeletal muscle cells, iron-induced ROS production accompanied by apoptosis is enhanced by ATG7 deficiency.	^237^
		VSMC-specific *Atg7*-KO mice suffer an elevation in aortic stiffness, and then compensatory mechanisms might be switched to maintain circulatory homeostasis.	^238^
		During the diabetic courses, AGEs are able to active vascular smooth calcification and apoptosis, which can be inhibited by ATG7 deficiency.	^239^
	Skeleton and Blood	Compared with WT mice, the osteoblasts-specific *Atg7*-KO ones have markedly lower relative bone formation rate. Furthermore, the interruption of autophagy causes a decrease in bone volume, thickness, stiffness, and ultimate breaking force.	^240^
		ATG7-deficient neutrophil precursors are observed to enhance glycolytic activity enhancement and lipid droplet accumulation, but impair mitochondrial respiration and reduce ATP production.	^241^
		ATG7 deletion in murine BM-MNCs has a negative effect on STAT3 activation accompanied by its nuclear translocation. ATG7-mediated autophagy can protect BM-MNCs from radiation-induced genotoxic stress.	^242^

*Atg7* autophagy-related gene 7.

Nineteen DNA sequence variants (DSVs) containing single-nucleotide polymorphisms (SNPs) have been observed in the *Atg7* gene promoter of coronary artery disease patients, including those with acute myocardial infarction (AMI). Multiple DSVs and SNPs alter the transcriptional activity of the *Atg7* promoter by affecting the combination of transcription factors and *Atg7* expression^16^. Genome-wide association studies and DNA methylation and exome sequencing data have identified 41 variants, 56 SNPs, and 38 SNPs of *Atg7* associated with HDL-cholesterol, systolic blood pressure, and blood pressure, respectively^17^. Moreover, *Atg7* variant rs8154 is a new prognostic marker for breast cancer based on in silico analysis^18^.

Parkinson’s disease (PD) is a fast-growing neurological disorder and neurodegenerative disease^19^. Reduced *Atg7* transcriptional activity was observed with four novel heterozygous variants and confirmed in five PD patients. Variations in the *Atg7* gene promoter alter ATG7 protein levels in PD patients and influence autophagic activity, possibly contributing to the onset of the disease^20^. Furthermore, elevated plasma ATG7 levels in the Han population of South China may result in susceptibility to late-onset sporadic PD^21^. An SNP in the *Atg7* gene can also impact the age of Huntington’s disease (HD) onset^22^. A European HD Network study demonstrated the impact of *Atg7* V471A polymorphism on the age of HD onset on Italians^23^.

#### Transcription factors and transcriptional regulators

The prominent transcription factor forkhead box O1 (FOXO1) is the main target of insulin signaling and is involved in interleukin-9 (IL-9) signaling pathways. FOXO1 is also a transcription-independent mediator of autophagy^24,25^. FOXO1 is acetylated by its dissociation from SIRT2, which determines its interaction with ATG7 and promotes autophagy under serum starvation^24,26^. The Ac-FOXO1-ATG7 complex activates autophagy independent of Beclin1 or Mechanistic target of rapamycin (mTOR)^24^. Under low oxidative stress (OS) conditions, FOXO1 maintains cell viability via its export from the nucleus, acetylation into Ac-FOXO1, and formation of the Ac-FOXO1-ATG7 complex. Under high OS levels, FOXO1 is located in the nucleus, promoting the transcription of proapoptotic proteins and apoptosis^27^. Most FOXO1 DNA-binding domain mutants preserve their interaction with cytoplasmic ATG7^28^. Moreover, the interaction between ATG7 and phosphorylated FOXO1 in the cytoplasm of immature natural killer (NK) cells is essential for NK cell maturation^29,30^. The FOXO1-ATG7 complex is also associated with bone formation and bone-related disorders^31^.

FOXO3, another *Atg7* transcriptional regulator, is predicted to bind *Atg7* at the ENCODE H3K4Me1 site 850 bp upstream of the *Atg7* transcription start site. FOXO3a-transactivated ATG7 acts as a tumor suppressor in non-small cell lung cancer (NSCLC) and mediates CK1α-induced autophagy, an anti-neoplastic mechanism^32^. Additionally, cellular redox imbalance and mitochondrial dysfunction in *Atg7*-KO mouse embryonic fibroblasts (MEF) cells are partly abrogated by FOXO1/3 overexpression through the restoration of antioxidant enzymes and reactive oxygen species (ROS) suppression^33^.

Heat shock factor 1 (HSF1) is a transcription factor and main regulator of temperature stress responses implicated in tumorigenesis^34^. HSF1 can directly bind to the promoter of *Atg7* to increase its expression, which is necessary for the cytoprotective autophagy induced by chemotherapeutic agents^35^. Furthermore, miR-217 induces HSF1/ATG7 pathway signaling by limiting NF1 expression and enhancing breast cancer cell autophagy, leading to chemoresistance^36^. During starvation, PSMD10 transfers to the nucleus and cooperatively binds to the *Atg7* gene promoter with nuclear HSF1 to upregulate ATG7 expression^37^. NBAT1 suppresses *Atg7* transcription by promoting PSMD10 degradation and inhibits PSMD10 and HSF1 occupancy on the *Atg7* promoter, inhibiting autophagy and chemoresistance in NSCLC^38^.

Transforming growth factor-β1 (TGF-β1) is a cytokine that increases the expression of autophagy-related genes, including *Atg7*^39^. TGF-β1 function is partly mediated by its regulation of Y-box binding protein 1 (YB-1), which binds to the *Atg7* promoter. The nuclear translocation of YB-1 induced by TGF-β1 promotes *Atg7* transcription and participates in the liver injury^40^. TGF-β1 is also a common fibrosis marker involved in multiple diseases. TGF-β1 and ATG7 play roles in fibrosis in many tissues in an autophagy-dependent manner. During maladaptive kidney repair, autophagy stimulates fibrogenesis by fibroblasts via the pro-fibrotic factor, fibroblast growth factor 2^41^. In contrast, ATG7-mediated autophagy suppresses vocal fold injury-induced fibrosis^42^. The TGF-β1/ATG7 axis is also involved in fibrosis in unilateral ureteral obstruction, SiO_2_-induced pulmonary inflammation, and radiation-induced skin injury, making autophagy a potential therapeutic target in fibrosis^43–45^.

Signal transducer and activator of transcription (STAT) 3 is activated by phosphorylation and forms homo- or heterodimers. It binds to the *Atg7* promoter, upregulating ATG7 expression. The IL-6/Janus kinase (JAK)/STAT3 pathway modulates skeletal muscle atrophy by regulating ATG7-mediated autophagy^46^. The inhibition of STAT3-mediated autophagy by ATG7 might be a novel target in triple-negative breast cancer^47^ and denervation-induced muscle atrophy^48^. Autophagy can increase the expression of pro-inflammatory cytokines, including IL-6 and IL-8^49,50^. These reports indicated that ATG7 might connect autophagy and inflammation.

Retinoid acid receptor is a transcription factor that can bind to the 5’-flanking region of the *Atg7* proximal promoter, regulating ATG7 transcription and hepatocellular carcinoma (HCC) progression^51^. CAMP response element-binding protein (CREB) is a transcriptional regulator that binds to the *Atg7* promoter at the −1809 to −1412 region. P38/Hsp27/CREB/ATG7 pathway signaling affects HCC chemoresistance by regulating autophagy^52^. Transcription factor NRF1 stimulates the *Atg7* promoter^53^, promoting autophagy in human nucleus pulposus cells^54^. Besides raising *Atg7* mRNA levels, EVI1 can also increase intracellular ROS levels involved in EVI1-induced autophagy. Therefore, EVI1 might promote drug resistance via dual control of ATG7^55^.

#### DNA methylation of *Atg7* gene

DNA methylation modulates multiple biological functions, including signal transmission, DNA repair, and gene expression^56^. Legionella Infection can irreversibly change the GATC motif to G(6 mA)TC in the *Atg7* promoter region, causing a time-dependent reduction in *Atg7* mRNA, which further inhibits autophagosome formation. These findings about host defense against Legionella, including autophagy, might help prevent and treat Legionnaires’ disease^57^.

*Atg7* was one of five hub DNA methylation-regulated genes in the Framingham heart study. Studies in monocytes and peripheral blood leukocytes showed that the *Atg7* methylation and expression status might be a novel epigenetic mechanism for coronary heart disease (CHD)^58^. The indirect methyltransferase inhibitor adenosine dialdehyde decreases *Atg7* expression and autophagy in cancer cells, indicating that *Atg7*-related methylation might be a potential target for treating breast and lung cancer^59^. Another study aimed at finding epigenetic alternations in miRNAs and DNA through an in silico approach identified *Atg7* as a hub gene and a potential target for gestational diabetes mellitus (GDM) diagnosis and treatment^60^.

### Regulation on *Atg7* mRNA

Modification and regulation of *Atg7* mRNA have been widely discussed in recent decades, including methylation and regulation by miRNA, lncRNA, circRNA, and RNA-binding proteins. The impact of these influential factors on *Atg7* can further affect multiple physiological processes and aging-related diseases (Table 2).

**Table 2:** Main regulatory mechanisms of *Atg7* at DNA and RNA levels

Regulation mechanism			Physiological processes and diseases	Reference
DNA level	G4-ligand	PDS	Neurodegeneration; Lewy Body disease	^14^
		BRACO19	Memory deficits	^15^
	Transcription factor	FOXO1	Bone formation; NK cell development, Innate immunity; Cancer treatment, including human cholangiocarcinoma, BC tumorigenesis	^26,29,31,243^
		FOXO3	An anti-neoplastic mechanism	^32^
		HSF1	Tumorigenesis	^35^
		TGF-β1	Fibrosis of many tissues, including liver, kidney, vocal fold, ureter, lung, and skin	^40–45^
		STAT3	Skeletal muscle atrophy	^46,48^
mRNA level	M^6^A-methylation		Osteoarthritis; Drug resistance	^63–65^
	miRNA	miR-129-5p	Heart failure	^68^
		miR-143, miR-182-5p, miR-20b-5p	Myocardial I/R injury	^69–71^
		miR-542-5p	H/R-induced cardiomyocyte injury	^73^
		miR-7-5p, miR-138-5p; MiR-1236-3p	DDP resistance	^80,81,83^
		miR-17	Glioblastoma development; Prostate cancer	^95,96^
		miR-20a	Glioma development; Prostate cancer	^95,97^
		miRNA-93a	Prolactinomas; Pituitary adenomas	^103^
		miR-106a	Colorectal cancer	^104^
		miR-210, miR-138-5p	Lung cancer	^106^
		miR-190A, miR-582-5p, miR-154	Bladder cancer	^108,109^
		miR-1275, miR-348	Breast cancer	^110^
		miR-202-5p, miR-106a-5p, miR-654-5p	DD/IDD	^116–118^
		miR-508-5p, miR-192-5p	AS	^119,120^
	lncRNA	IncRNA APF	Myocardial infarction	^75^
		lncRNA H19, lncRNA CHRF	Myocardial I/R injury	^69,70^
		LncRNA 0003250	Se deficiency	^79^
		lncRNA CCAT1, lncRNA DANCR, lncRNA TINCR	HCC	^92–94^
	circRNA	circRACGAP1	Gastric cancer	^87^
		circRNA0006948	Osteosarcoma	^144^
		circRNA0092276	Breast cancer	^110^
	RBP	HuR	renal tubular cell apoptosis	^146,147^
		LIN28A	Breast cancer	^148^

#### M^6^A-methylation

N6-methyladenosine (M^6^A) methylation is a common internal modification in eukaryotic mRNA^61^. M^6^A influences almost every fundamental aspect of mRNA metabolism and is controlled by RNA-binding proteins (RBPs), methyltransferase complexes, and demethylases. M^6^A targeting of *Atg5* and *Atg7* regulates adipogenesis by affecting autophagy. *Atg5* and *Atg7* are two potential targets of the fat-associated RNA demethylase, FTO^62^. Methyltransferase-like 3 (METTL3)-mediated M^6^A can attenuate *Atg7* mRNA stability, decreasing its expression and facilitating cellular aging and osteoarthritis by regulating the autophagy-GATA4 axis. Specifically, METTL3-mediated M^6^A initiates the decay of the *Atg7* transcript in a YTH M^6^A RNA-binding protein 2-dependent way^63^. Silencing METTL3 enhances *Atg7* mRNA stability and increases its expression. Synovium-targeted METTL3 siRNA downregulates senescence-associated secretory phenotype expression and increases autophagy in osteoarthritis-fibroblast-like synoviocytes, further alleviating cellular senescence in joints and destabilization of the medial meniscus-induced cartilage destruction^63^. METTL3 can regulate *Atg7* to reverse drug resistance in chronic myelocytic leukemia cells and gefitinib resistance in NSCLC cells caused by β-elemene^64,65^. In addition, *Atg7* M^6^A is increased in GDM with obesity^66^.

#### miRNAs

miRNAs belong to a highly conserved family that controls its downstream gene translation processes^67^. Several miRNAs have Inhibitory effects on *Atg7* in heart disease. Heart failure is a chronic disease associated with cardiomyocyte apoptosis and autophagy. MiR-129-5p inhibits autophagy and cardiomyocyte apoptosis, retarding heart failure progression^68^. MiR-143 expression relieves myocardial ischemia/reperfusion (I/R) injury by downregulating *Atg7* expression^69^. miR-182-5p and miR-20b-5p play a similar role^70,71^. Indeed, MiR-27a-5p could inhibit *Atg7* and had a cardioprotective effect against hypoxia-induced H9c2 cell injury, suggesting it is a potential strategy for healing hypoxia-related heart disease^72^. MiR-542-5p might also be a target for treating heart disease caused by hypoxia/reoxygenation-induced cardiomyocyte injury due to its relationship with *Atg7* mRNA^73^. Adipose-derived stromal cell-derived exosomes enhanced by miR-93-5p have cardioprotective effects after AMI in an *Atg7*-targeting pathway that inhibits autophagy and inflammatory response^74^. Similarly, miR-188-3p regulates autophagy and myocardial infarction by altering *Atg7* expression^75^. Moreover, atherosclerosis (AS) is also under the control of miR-188-3p by targeting *Atg7*^76^. MiR-221 negatively regulates FOXO3, inhibits *Atg7* transcription, and is a potential target for cardiac fibrosis after myocardial infarction (MI)^77^. The loss of cardiomyocytes after an injury, such as MI, might be compensated by cardiac progenitor cells; miR-143 participates in this process via targeting *Atg7*^78^. Studies in the Se-deficient chicken model showed that Se deficiency upregulates *Atg7* expression and inhibits cardiomyocyte autophagy by modulating miR-17-5p expression^79^.

Emerging studies have shown that miRNAs can directly inhibit *Atg7* in some key pathways involved in drug resistance in cancer. MiR-7-5p increases chemoresistance to cisplatin (DDP) in bladder cancer by suppressing invasion and inhibiting *Atg7* expression^80^. TRIM65 knockdown in A549/DDP cells downregulates autophagy and DDP resistance via the miR-138-5p/*Atg7* axis^81^. Similarly, miR-4486 inhibits autophagy and decreases DDP resistance in HCT116/DDP and SW480/DDP cells by targeting *Atg7*^82^. MiR-1236-3p also regulates autophagy and DDP resistance via its effects on *Atg7* mRNA expression^83^. MiR137 attenuates starvation-induced autophagy and promotes adriamycin sensitivity in U87 cells by regulating *Atg7* expression^84^. MiR-615-3p and miR-17 may regulate *Atg7* expression and further chemoresistance in NSCLC^85,86^. In addition, the miR-3657/*Atg7* axis increases the sensitivity of gastric cancer cells to apatinib^87^.

MiR-375 binds to a particular site within the 3’ untranslated region of *Atg7* mRNA, decreasing HCC cell viability under hypoxic conditions^88,89^. Additionally, autophagy in HCC cells is inhibited by miR-490-3p in an *Atg7*-targeting pathway^90^. Apigenin is a possible chemosensitizer in HCC and sensitizes BEL-7402/ADM cells to adriamycin via the miR-520b/*Atg7* axis^91^. The proliferation and invasion of HCC cells are similarly modulated by miR-181a-5p, miR-222-3p, and miR-375^92–94^. *Atg7* is a confirmed target of the miR-17 seed family, and *Atg7* inhibition by the miR-17 seed family (e.g., miR-20a and miR-17) has been reported in prostate cancer^95^. Furthermore, miR-17 negatively regulates *Atg7* expression and modulates autophagy in T98G glioblastoma cells^96^. Moreover, the viability of glioma cells is partly under the control of miR-20a^97^, while purple sweet potato delphinidin-3-rutin regulates the regulatory function of miR-20b-5p through the protein kinase B (AKT)/Creb/miR-20b-5p/*Atg7* pathway^98^.

MiR-186 can decrease *Atg7* and Beclin1 expression levels, thereby inhibiting autophagy in glioma-conditioned human cerebral microvascular endothelial cells^99^. MiR-96 in prostate cancer cells has a biphasic effect on autophagy by inhibiting mTOR or *Atg7*, which means the inhibition or ectopic overexpression both abolishes hypoxia-induced autophagy^100^. MiR-93 expression is negatively correlated with *Atg7* expression and increased in dopamine agonist-resistant prolactinomas^101^. MiRNA-93 downregulates *Atg7* and enhances cabergoline resistance of prolactinoma^102^. Along with clinical staging of neuroblastoma progression, miR-20a-5p expression decreases whereas its target *Atg7* increases^103^. MiR-106a can decrease *Atg7* levels and suppress the death of colorectal cancer cells^104^. MiR-210, an *Atg7*-targeting miRNA, can strongly enhance lung cancer cell proliferation, while another *Atg7*-targeting miRNA, miR-138-5p, inhibits the invasion and self-renewal of lung cancer stem-like cells^105,106^. MiR-190A, miR-582-5p, and miR-154 can increase the progression and drug resistance in bladder cancer cells by inhibiting *Atg7*^107–109^. Breast cancer is also regulated by *Atg7*-relevant miRNAs, including miR-1275 and miR-348^110^. Epithelial ovarian cancer (EOC) is regulated by *Atg7*-inhibiting miRNA, miR-6881-3p^111^. Other cancer types are also affected by the interaction between *Atg7* and miRNAs, including lymphoma via the hsa-miR-6511b-5p/*Atg7* axis^112^, pancreatic cancer (PC) through the miR-766-5p/*Atg7* axis^113^, retinoblastoma via miR-154-5p/*Atg7*^114^, and thyroid cancer through the miR-1343-3p/*Atg7* axis^115^.

MiRNAs also regulate *Atg7* in various aging-related diseases. A significant increase in miR-654-5p and its inhibition of *Atg7* were observed in degenerated nucleus pulposus tissues from intervertebral disc degeneration (IDD) patients^116^. In IDD nucleus pulposus, miR-202-5p overexpression reduces *Atg7* levels, while miR-202-5p inhibition enhances autophagy and reduces apoptosis in nucleus pulposus cells^117^. Melatonin enhances *Atg7* transcription and translation by inhibiting miR-106a-5p in annulus fibrosus cells in disc degeneration patients^118^. MiR-192-5p reduction increases *Atg7* expression and inhibits vascular smooth muscle cell (VSMC) proliferation and migration. Therefore, serum miR-192-5p may be a novel diagnostic biomarker for atherosclerosis (AS)^119^. Another related miRNA, miR-508-5p, inhibits *Atg7* expression and blocks autophagy in endothelial cells, which is important for AS procession^120^. In addition, the interactions between miRNAs and *Atg7* may regulate ischemic brain injury^121^, cerebral I/R injury^122,123^, neuronal apoptosis^124^, *M. tuberculosis* infection^125,126^, allergic asthma^127^, systemic lupus erythematosus^128^, sepsis^129^ and sepsis-induced acute kidney injury (AKI)^130^, nonobstructive azoospermia^131^, urethral stricture and urethral fibrosis^132^, hypoxia-induced pulmonary hypertension^133^, hepatic I/R injury^134^, hepatitis B virus replication^135^, inflammatory bowel disease^136^, diabetes mellitus^137^, pancreatic ductal adenocarcinoma (PDAC)^138^, and senile cataracts^139^.

#### LncRNAs

LncRNAs are long-stranded noncoding RNA with a length greater than 200 nucleotides. LncRNA exerts various effects by binding to DNA, RNA, and proteins^140^. LncRNA APF regulates autophagy and myocardial infarction by targeting miR-188-3p, which directly binds with *Atg7*^75^. Autophagy mediated by the lncRNA H19/miR-143/*Atg7* signaling axis relieves myocardial I/R injury under 6-Gingerol treatment conditions^69^. In addition, lncRNA CHRF enhances autophagy and exacerbates myocardial ischemia/reperfusion injury by modulating the miR-182-5p/*Atg7* pathway^70^. LncRNA TUG1, which is induced by E26 transformation-specific proto-oncogene 2 (ETS2), sponges miR-129-5p to increase *Atg7* expression. *Ets2*-KO inhibits *Atg7* mediate-autophagy and postpones the development of heart failure^68^. LncRNA 0003250 regulates miR-17-5p, which alters the *Atg7* expression and is correlated with Se deficiency^79^. Multiple molecular pathways in HCC are regulated not only by miRNA but also by upstream lncRNAs. The proliferation and invasion of HCC are regulated by lncRNA TINCR, which modulates the miR-375/*Atg7* axis^94^. LncRNA DANCR sponges miR-222-3p and promotes *Atg7* expression to accelerate HCC proliferation and autophagy^93^. Similarly, lncRNA CCAT1 regulates *Atg7* by sponging miR-181 in HCC^92^.

LncRNA RASSF8-AS1 regulates autophagy by targeting miR-188-3p, which increases *Atg7* levels and may be a novel target for the prevention and prognosis of AS^76^. The lncRNA nicotinamide nucleotide transhydrogenase-antisense RNA1 via miR-1236-3p/*Atg7* axis increases DDP resistance in lung cancer^83^. Many other physiological processes are regulated in this manner, such as the lncRNA SNHG8/miRNA-588/*Atg7* axis in colorectal cancer^141^, lncRNA HULC/*Atg7* axis in epithelial ovarian carcinoma^142^, lncRNA PVT1/miR-186/*Atg7* axis in glioma vascular endothelial cells^123^, lncRNA GAS8-AS1/miR-1343-3p/*Atg7* axis in thyroid cancer^115^, lncHOTAIR/hsa-miR-6511b-5p/*Atg7* axis in lymphoma^112^, lncHOTAIR/miR-20b-5p/*Atg7* axis in hepatic I/R injury^134^, lncRNA SNHG3/miR-485/*Atg7* axis in brain I/R injury^123^, Lnc-FSD2-31:1/miR-4736/*Atg7* axis in PDAC^138^, and lncRNA WAC-AS1/miR-192-5p/*Atg7* axis in hepatitis B virus replication^135^.

#### CircRNAs

CircRNAs are novel RNA molecules characterized by covalently closed loops widely present in eukaryotes^143^. In EOC, circ-EEF2 promotes autophagy by interacting with miR-6881-3p and upregulating its target *Atg7*^111^. CircRNA RACGAP1 is a sponge for miR-3657 to upregulate *Atg7* expression, and circRACGAP1 knockdown increases the sensitivity of gastric cancer cells to apatinib^87^. CircRNA0006948 modulates *Atg7* expression levels through the CircRNA0006948/miR-490-3p/*Atg7* pathway, which regulates the invasion, proliferation, and migration of osteosarcoma^144^. CircRNA0092276 regulates the miR-348/*Atg7* axis to promote autophagy and the doxorubicin resistance in breast cancer^110^. Autophagy-associated circAtg7 is located in both the nucleus and cytoplasm. Nuclear circAtg7 is a scaffold that strengthens the interaction between *Atg7* mRNA and human antigen R protein to stabilize *Atg7* mRNA. Cytoplasmic circAtg7 is a sponge for miR-766-5p that increases *Atg7* expression. Furthermore, circAtg7 regulates PC cell proliferation and metastasis by modulating autophagy^113^.

*Atg7* is involved in other pathways, including the chr10:115386962-115390436+/miR-6914-5p/*Atg7* axis in inflammatory bowel disease^136^, circ-ADAM9/miR-20a-5p/*Atg7* axis in diabetes mellitus^137^, circHIPK3/miR-190b/*Atg7* axis in atherosclerosis^145^, circPAN3/miR-221/*Atg7* axis in cardiac fibrosis^77^, circHIPK3/miR-20b-5p/*Atg7* axis in myocardial I/R injury^71^, and Hsa_circ_0004058/miR-186/*Atg7* axis in senile cataract^139^.

#### Others

RBPs interact with RNA directly via specific RNA-binding domains and participate in many post-transcriptional regulatory processes. The RBP HuR binds to the coding region of *Atg7* mRNA to regulate hypoxia-induced autophagy^146^. Additional mechanistic studies showed that HuR increases *Atg7* mRNA stability by binding to its AU-rich elements^147^. This interaction mainly occurs in patients with diabetic intervertebral disc degeneration or under hypoxia conditions^146,147^. RBP LIN28A also interacts with *Atg7* mRNA, which might be the target for LIN28A regulation at the post-transcriptional level. This interaction can increase *Atg7* mRNA stability and protein levels and promote chemoresistance in breast cancer^148^.

U2AF35 belongs to the splicing factor SR gene family, which is frequently mutated in various diseases. Recurrent mutations in U2AF35 (S34F) have been observed in myelodysplastic syndrome and tumors and promote transformation by generating aberrant *Atg7* pre-mRNA 3’ ends. In U2AF35(S34F)-transformed cells, the *Atg7* pre-mRNA is processed incorrectly, leading to secondary mutations^149^.

### Post-translational modifications of the ATG7 protein

ATG7 can be modulated during autophagy by several post-translational modifications, including acetylation, deacetylation, and ubiquitination.

#### Acetylation and deacetylation

The p300 acetyltransferase regulates the acetylation of various autophagy proteins. Disruption of p300 can reduce ATG7 acetylation and stimulate autophagy, while p300 overexpression increases ATG7 acetylation and inhibits starvation-induced autophagy^150^. BCL2-associated athanogene 6 (BAG6)/HLA-B-associated transcript 3 increases p53 acetylation and limits the p300-dependent acetylation of ATG7^151^. Additionally, BAG6 tightly controls p300 intracellular localization, influencing the accessibility of p300 to ATG7 and p53 and controlling autophagy^151,152^. Under various pathophysiological conditions, the beta chain of the non-classical MHC-II protein HLA-DM restricts the human T-cell leukemia virus type-1 (HTLV-1) expression by directly interacting with ATG7 and regulating ATG7 acetylation promoted by p300^153^. A histone acetyltransferase encoded by the M. oryzae ortholog of GCN5 acetylates ATG7, downregulating light- and nitrogen-starvation-induced autophagy in response to important environmental changes^131^.

The NAD-dependent deacetylase Sirt1 regulates autophagy and the cellular starvation response^154^. Sirt1 mediates chondrocyte autophagy by physically interacting with and deacetylating ATG7^155^. Osteoarthritis (OA) is an age-related degenerative disorder accompanied by pain and joint mobility disturbances. Sirt1 levels decrease in aged or OA cartilage compared to young and healthy cartilage. Sirt1 deacetylates the lysine residues on ATG7 and other crucial autophagy proteins to increase autophagy in chondrocytes. Chondrocyte-specific Sirt1 silencing decreases chondrogenic markers. Thus, Sirt1 is a potential target for OA intervention^156^. Aldehyde dehydrogenase 2 (ALDH2)-enhanced Sirt1 modulates the interaction between LC3 and ATG7. ALDH2 activation in aging hearts enhances LC3 deacetylation in the nucleus and the formation of LC3-ATG7 complexes in the cytoplasm by controlling Sirt1 activation and nuclear localization^157^.

**Fig. 3:**
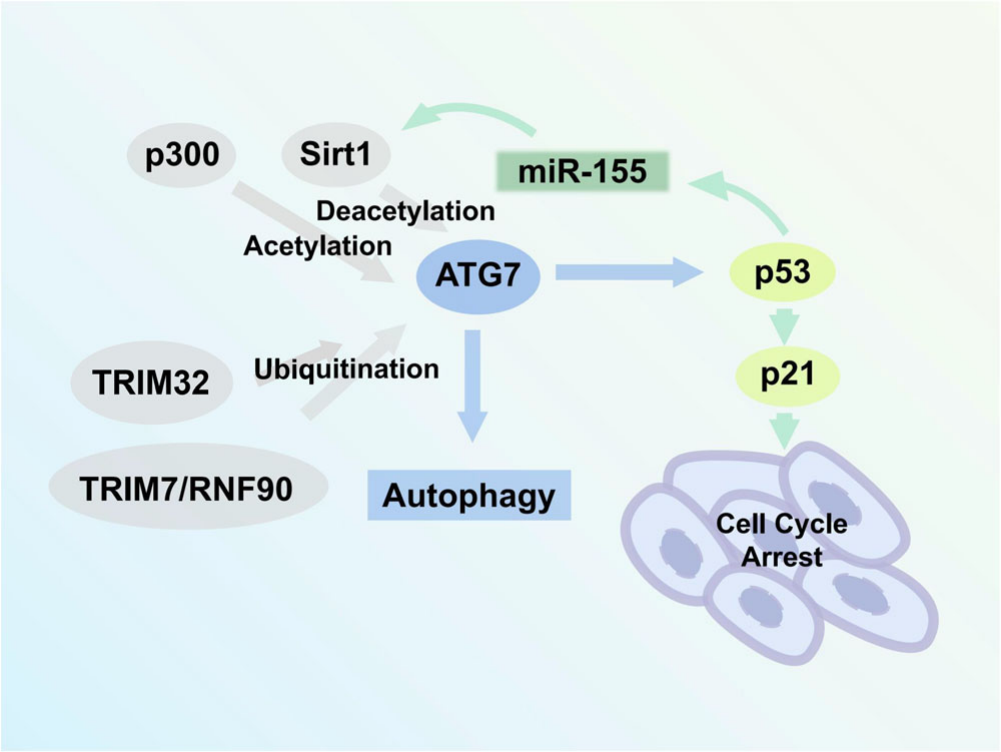
p53/Sirt1/ATG7 axis and ATG7 epigenetic modification. It has been reported that ATG7 is regulated by epigenetic modifications of acetylation, deacetylation, and ubiquitination. p53/miR-155/Sirt1 axis forms a autophagy-regulating loop with ATG7.

ATG7 is also involved in the Sirt1/p53 signaling pathway. As a transcription factor, p53 regulates miR-155 expression, and miR-155 directly targets the Sirt1 3’UTR region. Under high-glucose conditions, the p53/miR-155/Sirt1/ATG7 mediates autophagy and could be a potential therapeutic target for diabetic kidney injury^158^ (Fig. 3).

#### Ubiquitination

The ubiquitination of proteins plays a role in proteasome degradation, immune response regulation, mitochondrial autophagy, epigenetic regulation, and apoptosis^159^. Recent studies have indicated that the RING-type E3 ligase, tripartite motif-containing protein (TRIM) 7/RNF90, promotes ATG7 ubiquitination. TRIM7 is a core factor in infection-, starvation- and rapamycin-induced autophagosome accumulation. It helps host cells resist bacterial infection by inducing the K63-linked ubiquitination of ATG7 at K413 to positively regulate autophagy. Moreover, experiments in mouse models have shown that ATG7 ubiquitination at K413 or K409 has a decisive impact on *L. monocytogenes* infection and host cell resistance. Autophagosome accumulation after *L. monocytogenes* infection of wild-type cells but not cells with TRIM7 or ATG7 inactivating mutations clears intracellular bacteria and exerts defensive effects^160^.

Oxidative stress-induced autophagy helps prevent cell damage and maintains cellular homeostasis^161^. Previous studies have shown that ROS activates the ataxia-telangiectasia mutated (ATM)-cell cycle checkpoint kinase 2 (CHK2) pathway and promotes autophagy. When the ATM/CHK2 pathway is activated by ROS, ATM and CHK2 phosphorylation leads to TRIM32 phosphorylation at S55, activating its E3 ubiquitin ligase function. TRIM32 activation causes ATG7 K63-linked ubiquitination at K45, initiating ROS-induced autophagy. In addition, Liu et al. showed that the ATM/CHK2/TRIM32/ATG7 axis might alleviate I/R injury in mice by activating autophagy^162^.

## ATG7 functions and its relationship with aging-related diseases

ATG7 plays a crucial role in two ubiquitin-like conjugation systems involving ATG12 and LC3^163^. The ubiquitin-like conjugation system involving ATG12 also contains ATG7, ATG5, ATG16, and ATG10; the ATG12-ATG5-ATG16 complex forms an E3 ligase^6,7^. ATG7 catalyzes another ubiquitin-like conjugation system with ATG3 and ATG4 for the lipidation of LC3^164^. On this basis, the classical autophagy function and related regulatory mechanism of ATG7 are involved in several physiological processes and aging diseases. Several studies have indicated that ATG7 has additional functions beyond autophagy.

### Autophagy-dependent functions of ATG7

#### Cell fate

Theoretically, autophagy and apoptosis are crucial catabolic processes regulating cell maintenance and tissue homeostasis^165^. ATG7 promotes apoptosis, prevents epithelia-mesenchymal transition (EMT), and inhibits proliferation by blocking aerobic glycolysis in the mitochondria of triple-negative breast cancer (TNBC) cells^166^. Studies with human periodontal ligament cells also demonstrated that ATG7 overexpression contributes to the proliferation and inhibits apoptosis in aging cells^167^. Autophagy is renoprotective during AKI, especially in vancomycin (Van)-induced AKI^168^. Researchers found that the cell death-related gene, PKC-δ, was markedly suppressed in the *Atg7*-KO model after Van treatment. ATG7 might induce renal cell apoptosis after Van treatment by binding to PKC-δ^168^.

TNFAIP8 interacts with the ATG7-ATG3 complex and facilitates LC3 lipidation. The TNFAIP8-ATG7 interaction is important in regulating autophagy and proliferation in liver cancer cells^169^.Alzheimer’s disease (AD) is a common neurodegenerative disorder mainly caused by β-amyloid (Aβ) peptide accumulation. Estrogen receptor β can promote autophagy by interacting with ATG7, which further improves the autophagy-lysosomal activity in Aβ clearance and decreases the risk of AD^170^.

ATG7 regulates the cell cycle and thrombopoiesis during hematopoiesis, counteracting hematopoietic aging and leukemogenesis at multiple stages. ATG7-dependent canonical autophagy is critical for hematopoietic stem cells, but not for differentiated or somatic cells^171^. *Atg7*-KO mice are more prone to engrafted leukemogenesis, demonstrated by increased white blood cells, lymphocytes, and platelets. ATG7 deletion causes the deterioration of hematopoietic stem and progenitor cell (HSPC) function, which may lead to leukemogenesis^172^. Additional mouse studies revealed that the loss of ATG7 in HSPCs led to a lethal pre-leukemic phenotype^173^. Autophagy is implicated in the cell cycle of HSPCs in a nutrient-dependent manner; the absence of ATG7 leads to the ablation of the HSCPs cell cycle^174^. Transcriptomic analysis of HSPCs suggested that hematopoietic ATG7-related autophagy defects caused elevation of iron activity, inhibited osteocyte differentiation and calcium metabolism, potentially leading to bone loss and osteoporosis^175^. *Atg7*-deficient models had mitochondrial and cell cycle dysfunction, impaired platelet production, and failed hemostasis^176^. In addition, abolishing ATG7-related autophagy in the hematopoietic system greatly impacts the aging of non-hematopoietic organs^177^.

Reduced osteoblast differentiation is critical in bone-related pathogenesis, including OA and osteoporosis. Ferutinin significantly increases the expression of KLF2, ATG7, and several other autophagy-related proteins in dental pulp-derived stem cells. In contrast, *Atg7*-or *Beclin1*-KO reduces KLF2 and the levels of osteoblast (OB) differentiation-related molecules, indicating that ferutinin regulates OB differentiation via KLF2 and autophagy^178^. ATG7 and superoxide dismutase 2 are involved in the pathogenesis of osteoporosis, and their expression levels correlated in osteoporotic mice and osteoporosis-free mice^179^. Another study using dexamethasone-induced osteoporotic mouse models suggested that glucocorticoid-induced bone loss enhanced ATG7-related autophagy in osteoclasts via the PI3K/Akt/mTOR signaling pathway^180^.

#### Cell structure and development

The lamellar body (LB) is a specific lysosome-related organelle in type 2 alveolar epithelial cells, specifically related to autophagy^181,182^. *Atg7*-KO in mice interrupts the maturation process of LB and the production of surfactant protein B, indicating that ATG7 is necessary for the formation, maturation, and maintenance of LB^183^. In addition, suppression of AMP-activated protein kinase-mediated autophagy leads to poor lung development or even bronchopulmonary dysplasia^184^.

Increased ATG7 in neural tubes promotes neural crest cell delamination by suppressing the BMP4/Smad signaling pathway. Moreover, ATG7 overexpression accelerates cell progression toward S phase^185^. Additionally, research using ATG7 heterozygous mice suggested that ATG7 is involved in progeria by modulating autophagy^186^.

ATG7 regulates cerebrovascular development by promoting endothelial fibronectin expression and modulating Protein kinase A activity. *Atg7*-endothelial knockout (eKO) reduces fibronectin expression and causes cerebral astrocyte-microvascular disassociation, impairing the blood-brain barrier homeostasis^187^. ATG7 deletion leads to frustration in epinephrine-stimulated von Willebrand factor (VWF) secretion accompanied by prolonged bleeding time. Moreover, the processing, maturation, and secretion of VWF in endothelial cells is controlled by ATG7-mediated autophagy^188^. In chick embryos, ATG7 expression is observed in the plexus vessels of angiogenesis, and interruption of autophagy blocks angiogenesis by altering cell viability and migration^189^. Ethanol exposure can enhance autophagy by upregulating LC3 and ATG7 expression levels. After ethanol treatment, the incidence rate of congenital cardiovascular diseases in chick embryos increases^190^ High-glucose levels increase ATG7 and LC3 expression, indicating that autophagy may also be involved in high-glucose-induced cardiovascular malformation^191^.

ATG7 prevents EMT by suppressing aerobic glycolysis^166^. During EMT, epithelial cells lose their cell polarity, resulting in higher mesenchymal phenotypes that might affect the progression of malignant tumors derived from epithelial cells^192^. Altered ATG7 expression levels correlate with tumor progression and prognostic outcomes in TNBC^166^. In *Atg*7-KO retinal pigment epithelium (RPE) cells, the epithelial marker claudin-1 is reduced, while mesenchymal markers are decreased, leading to cell migration and enhanced contractility. ATG7-mediated autophagy is a notable mechanism in EMT resistance^193^. Research with chicken embryos has shown that ATG7 affects whole embryonic development by regulating EMT. During EMT in gastrulation, ATG7 expression is observed on the top of the endoderm and ectoderm. E-cadherin, a marker of EMT, increases when ATG7 is overexpressed^194^.

#### Others

It has been reported that ATG7(−/−) mitochondria have deficiencies in mitochondrial respiration. Reduced resting mitochondrial oxygen consumption, increased compensatory basal glycolytic rates, and increased steady-state ROS levels have been observed in ATG7(−/−) cells. Pancreatic beta cell-specific *Atg7*-KO mice exhibit mitochondrial dysfunction along with oxidative stress, indicating the important role of ATG7 in glucose metabolism^195^. Impaired ATG7-mediated autophagy enhances muscle loss and sarcopenia in the aging population, and betaine can regulate autophagy to stop degeneration in aged muscle^196^. Moreover, a proteotoxic heart failure study suggested that ATG7-induced autophagy could decrease cardiac hypertrophy and interstitial fibrosis, ameliorate ventricular dysfunction, and reduce intracellular aggregates^197^. In addition, the regulation of nuclear factor-kappaB (NF-κB) by ATG7 can also affect drug resistance in cancer cells. The blockade of ATG7-mediated autophagy interrupts IκB degradation by inhibiting cathepsin D, which further activates the NF-κB signaling pathway, which can influence the drug sensitivity of cancer cells^198^.

### Autophagy-independent functions of ATG7

#### Cell fate

The transcription factor p53 is a key tumor suppressor that plays fundamental roles in cancer immunity and inflammation^199^. It is also involved in many cellular functions, including metabolism, DNA repair, cell cycle arrest, cell differentiation, senescence, and cell death^200^. Previous studies have shown a significant relationship between p53 and autophagy-related proteins, especially ATG7^201,202^. The absence of ATG7 can increase mitochondrial ROS and DNA damage, affect cell cycle inhibitor p21 expression, and reciprocally regulate p53-dependent cell cycle and cell death pathways by activating p53. These observations demonstrated reciprocal regulation in which ATG7 can bind to and regulate p53 and further modulate the cell cycle and survival during metabolic stress^203^. Cnot-3, a component of the CCR4-NOT complex, can shorten the poly(A) tail of *Atg7* mRNA. ATG7 and p53 co-immunoprecipitate from *Cnot3*-KO heart lysates, showing that these two proteins interact. Additional research indicated that the nuclear ATG7 and p53 levels increased in *Cnot3*-KO cells compared to wild-type cells, and nuclear ATG7 modulated p53 activity to induce the expression of cell death-promoting factors^204^. A recent study showed that ATG7, p53, and VIM3 form a complex in prostate cancer and BPH-1 cells. Moreover, the VIM3/p53/ATG7 complex affected the migration of prostate cancer cells by binding with the pri-miR-371a-3p promoter, providing a novel method of prostate carcinoma differentiation^205^.

#### Metabolic reprogramming

The Warburg effect is a special energy metabolic process first described in cancer cells. This effect is frequently found in cancer tissues and many other rapidly dividing normal cells^206^. Autophagy provides a mechanism by which cells can cope with energy crises and is associated with energy metabolism in tumor cells. ATG7 inhibits the Warburg effect by binding PKM2 and preventing its phosphorylation on Tyr-105 by FGFR1^207^. Loss of ATG7 produces the opposite result, promoting EMT in tumor cells. The link between ATG7 and the Warburg effect could provide new strategies for cancer treatment^207^.

#### NF-κB

Research has shown that ATG7 exerts its effects by regulating transcription. The proangiogenic activity of ATG7 in the brain is mediated by IL-6 production, which is dependent on NF-κB^208^. *Atg7*-eKO can alleviate brain inflammatory responses after I/R in mice. *Atg7*-eKO reduces IKKβ phosphorylation, which inactivates NF-κB and decreases the mRNA levels of several pro-inflammatory cytokines, including IL-6 and IL-8. Interestingly, transcriptional regulation by ATG7 is independent of its role in autophagy^209^.

Inflammation and ATG7 have a complicated relationship in bone disease. Inflammation inhibits autophagy, proliferation, and the cell cycle in chondrocytes, manifested by arrest in G1, a reduced S phase, and downregulation of ATG7, ATG5, p62, and LC3. Inhibiting the PI3K/AKT/mTOR signaling pathway relieves inflammatory responses in OA rat models^210^. Increased Grb2-associated-binding protein-2 (GAB2) in chondrocytes correlates with OA progression in in vivo and in vitro OA models. Furthermore, GAB2 inhibition decreases p62 expression but increases the expression of ATG7 and other autophagy-related proteins^211^.

#### Others

ATG7 is also essential for counteracting hematopoietic aging. Knocking out *Atg7* or *Atg5* showed that ATG7 plays a non-autophagic role in maintaining normal nucleosome assembly and decelerating aging in the CD11b(+)Ly6G(−) cell population in the bone marrow. ATG7 deletion significantly confers an aging phenotype on this blood lineage subgroup. These results indicate a dual role for ATG7 in resisting hematopoietic aging^212^. In addition, ATG7 promotes OCT4 transcription and stem-like properties by interacting with and stabilizing β-catenin. This mechanism may promote the cancer stem-like cell characteristics of prostate cancer, including tumor initiation, self-renewal, and drug resistance^213^.

## Summary and future directions

ATG7, an E1-like ubiquitin-activating enzyme, promotes the formation and extension of autophagosome membranes, thus initiating cellular autophagy. The basic autophagy-dependent functions of ATG7 involve two ubiquitin-like conjugation systems—ATG12-ATG5 and LC3-phosphatidylethanolamine. Besides its E1-like effects in autophagy, whether ATG7 could serve as an E1 enzyme in ubiquitination reactions needs to be clarified.

**Fig. 4:**
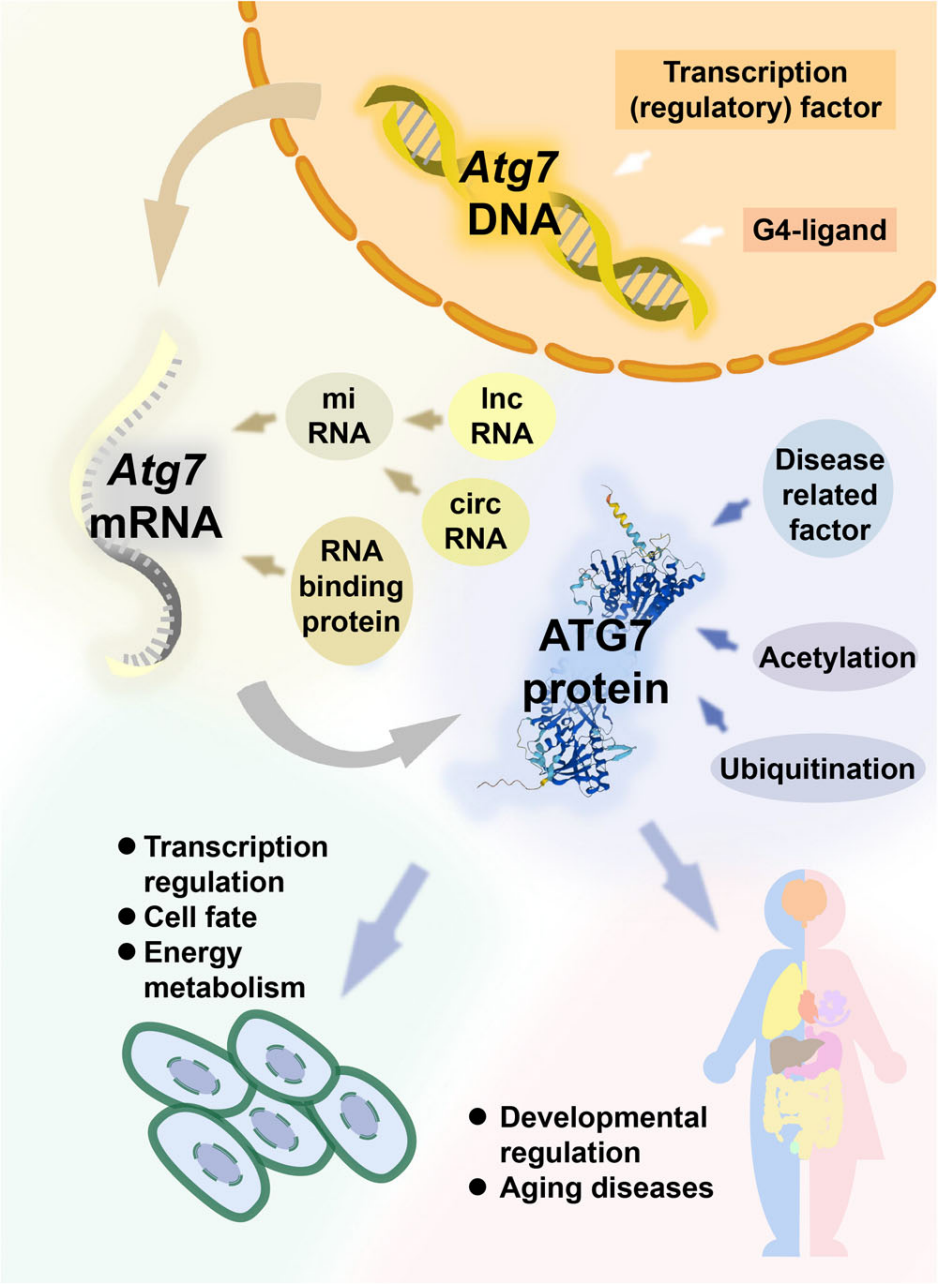
An overview of ATG7 function and regulatory mechanism. ATG7 is regulated at DNA, mRNA, and protein levels, and is closely associated with transcription regulation, cell fate, energy metabolism, developmental regulation and aging diseases.

ATG7 protein is involved in many aging diseases and could serve as a promising therapeutic target (Fig. 4). Importantly, ATG7 plays different roles in various tissues. Muscle-specific deletion of ATG7 downregulates muscle mass and strength and causes abnormal mitochondria and swollen sarcoplasmic reticulum. Excessive activation of ATG7-mediated autophagy also induces severe muscle loss^214^. VSMC-specific *Atg7*-KO causes sarcoplasmic reticulum swelling and imbalanced Ca^2+^ homeostasis, leading to altered contractility^215^. In colon epithelial conditional *Atg7*-KO mice, experimental colitis deteriorated, with more bacterial intrusion into the colonic epithelium. Furthermore, the expression of antimicrobial or antiparasitic peptides and the secretion of colonic mucins were diminished in the conditional *Atg7*KO mice, leading to abnormal microflora and colitis^216^. Mice deficient for pancreatic ATG7 suffered earlier death caused by inflammation, fibrosis, increased apoptosis and necroptosis, and declining exocrine and endocrine functions^217^. Experiments in the drosophila eye revealed that both ATG7 and Hsp27 are involved in normal eye development. Overexpression of ATG7 could rescue Hsp27-deficient phenotypes; however, overexpression of Hsp27 could not rescue ATG7-deficient phenotypes^218^. ATG7 also participates in liver pathology and the adipose-liver system. Adipocyte-specific loss of *Atg7* enhances high-fat diet (HFD)-induced inguinal white adipose tissue hypertrophy, which downregulates serum-free fatty acid levels and relieves HFD-induced steatosis, liver inflammation, and fibrosis through adipose-liver crosstalk^219^. In certain diseases (e.g., AKI), activating ATG7-mediated autophagy might alleviate the symptoms. Understanding the various roles of ATG7 in different tissues could shed new light on targeting ATG7 to modulate diverse tissue dysfunctions.

Multiple genetic and transcriptional mechanisms regulate the autophagy-dependent functions of ATG7. Transcription factors, transcriptional regulatory factors as well as miRNA, circRNA, lncRNA, and RBPs regulate *Atg7* mRNA expression. Moreover, various post-translational modifications regulate ATG7 protein function. Although multiple regulatory mechanisms have been defined for the autophagy-dependent functions of ATG7, how the autophagy-independent functions of ATG7 are regulated remains unclear. Because ATG7 might be regulated differently when performing autophagy-dependent and autophagy-independent functions, it is important to determine how ATG7 switches between its autophagy-dependent and autophagy-independent roles.

DNA methylation can control genetic expression without altering DNA sequence by changing chromatin structure, DNA conformation, DNA stability, and DNA-protein interactions. Until recently, little was known about the DNA methylation of *Atg7* and its functions. One study reported that *Atg7* gene methylation can be affected by Legionella Infection, which causes a reduction in *Atg7* mRNA^57^. The studies in monocytes and peripheral blood leukocytes showed that the methylation status and expression status of *Atg7* might clue a novel epigenetic mechanism for CHD^58^. The epigenetic modifications of *Atg7*, including DNA methylation, require further study. Methylation is an important post-translational modification that adds methyl groups to amino acid residues in proteins, thereby altering protein structure and function. Methylation of a protein can regulate its stability, localization, and interactions, significantly affecting biological processes, including gene expression and cellular signaling. The methylation and other possible post-translational modifications of ATG7 protein are likely to have significant implications, and further research in this field is needed.

Along with the improvement of technologies and a comprehensive understanding of ATG7 regulatory mechanisms, it is anticipated that targeting ATG7 could benefit aging-related diseases. Future research should elucidate the specific molecular mechanisms underlying how ATG7 exerts its multiple functions and how these functions are related to aging diseases and corresponding therapies.

## Data Availability

All data are available online.
